# Case Report: Fatal Outcome for a Preterm Newborn With Meningitis Caused by Extended-Spectrum β-Lactamase-Producing *Escherichia coli* Sequence Type 1193

**DOI:** 10.3389/fped.2022.866762

**Published:** 2022-04-06

**Authors:** Frida Oldendorff, Agnes Linnér, Mikael Finder, Peter Eisenlauer, Malin Kjellberg, Christian G. Giske, Viveka Nordberg

**Affiliations:** ^1^Astrid Lindgren Children’s Hospital, Karolinska University Hospital, Stockholm, Sweden; ^2^Department of Neonatology, Astrid Lindgren Children’s Hospital, Karolinska University Hospital, Stockholm, Sweden; ^3^Department of Women’s and Children’s Health, Karolinska Institutet, Stockholm, Sweden; ^4^Department of Clinical Science, Intervention and Technology (CLINTEC), Division of Paediatrics, Karolinska Institutet, Stockholm, Sweden; ^5^Department of Laboratory Medicine, Division of Clinical Microbiology, Karolinska Institutet, Stockholm, Sweden; ^6^Department of Clinical Microbiology, Karolinska University Laboratory, Stockholm, Sweden

**Keywords:** neonatology, meningitis, extended-spectrum β-lactamase, sepsis, case report

## Abstract

**Introduction:**

In this case report, we describe an extended-spectrum beta-lactamase (ESBL) – *Escherichia coli (E. coli)* strain of sequence type (ST) 1193, a novel, virulent, multidrug-resistant (MDR) clone with a rapid global spread. ST 1193 has been more commonly associated with invasive disease than other ESBL-*E. coli* STs. To our knowledge, this is the first known case in Sweden where a newborn died of an ESBL-*E. coli* ST 1193 meningitis. We emphasize that the clinical knowledge about the properties of certain MDR-clones should be increased.

**Case Report:**

A moderately preterm boy was born after preterm prolonged rupture of membranes. The mother had an ESBL-*E. coli* urinary tract infection during pregnancy. At 36 h of age he developed signs of infection and was given first-line therapy for early onset sepsis. Thereafter he developed seizures. The treatment was changed to cover suspected meningitis. Culture showed growth of the same ESBL- *E. coli* ST 1193 strain in the child’s blood and cerebrospinal fluid, as well as in the mother’s urine. Antibiotics were adapted. His condition deteriorated and he developed fulminant septic shock with treatment-resistant seizures. The boy passed away at 3 days of age.

**Conclusion:**

This case highlights the risk of delay in diagnosis when a marking for carriage of MDR-bacteria is falsely removed from a medical record of a pregnant women. Further, it demonstrates that ESBL-*E. coli* ST 1193 infection in neonates can be fatal. Thus, studies regarding virulence factors of ESBL-*E. coli* infections in pregnant women and their children are needed to understand the association between this infection and severe invasive disease in newborn children.

## Introduction

Neonatal meningitis caused by Gram-negative bacteria (GNB) is a feared condition with a high mortality and morbidity rate, with *Escherichia coli (E. coli)* being one of the most common causative bacteria (1, 2). *E. coli* strains are divided into four phylogroups according to their virulence determinants, where phylogroup B2 is associated with invasive disease such as pediatric sepsis and meningitis (3, 4). Further, there is evidence of a higher mortality in newborns with sepsis caused extended-spectrum beta-lactamase (ESBL)-producing GNB, compared to non-resistant strains (5). In our case of neonatal meningitis, the *E. coli* strain was seen to belong to the phylogroup B2 and sequence type (ST) 1193. *E. coli* ST 1193, first described in 2012, is a virulent, multi drug resistant (MDR) clone with characteristics that have contributed to its rapid global spread and a high capacity for invasiveness, which belongs to a previously unknown clonal complex (cc) (6–8). To our knowledge, this is the first fatal case of ESBL-*E. coli* ST 1193 neonatal meningitis in Sweden.

Individuals carrying MDR may be affected by the carriage to a greater or lesser extent depending on the virulence factors carried by the strain. Virulence factors contribute to the strain’s greater propensity to cause local or invasive infection and to colonize the gut for longer or shorter time periods (9). It has been shown that gut colonization of community-acquired strains does not cause infection as frequently as virulent strains from hospital settings (10). Moreover, a high degree of maternal-neonatal transmission of MDR has been seen, as well as an increasing MDR prevalence in the community (11–13). In Sweden no molecular characterization is presently carried out of ESBL-*E. coli* isolated from the urine of pregnant women.

## Case Report

**FIGURE 1:**
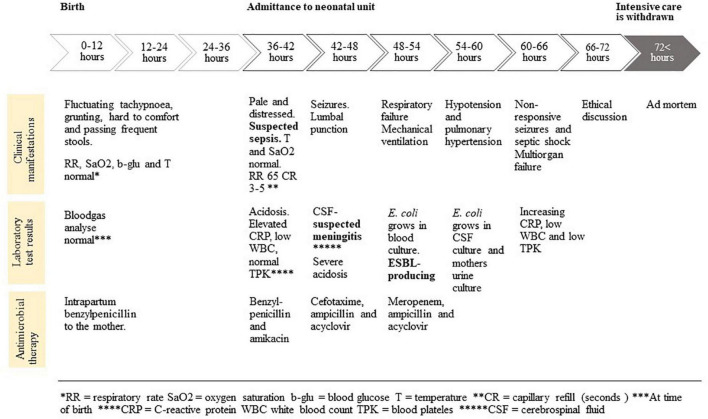
Timeline.

This case describes a male patient born vaginally at 36 weeks + 2 days after preterm prolonged rupture of membranes (32 h) with a birthweight of 2.8 kg and Apgar score of 9-10-10. Umbilical cord blood-gas analysis, performed after birth, showed normal potential of hydrogen (pH), base excess (BE), and lactate, see Timeline (Figure 1). The pregnancy had been normal, apart from several urinary tract infections (UTIs) during the second trimester. The UTIs had been treated by the primary health-care center, where growth of ESBL-*E. coli* in the urine had been detected and the mothers medical record had been marked to signal colonization with MDR bacteria. Further molecular characterization was not performed, as it is not routine on ESBL-*E. coli* isolates from urine. After treatment with pivmecillinam twice and nitrofurantoin twice, a control culture of the mother’s urine was negative, which led to the removal of the warning in the medical record by the treating physician. At time of delivery, the mother was given benzylpenicillin intrapartum, as per routine in case of prolonged rupture of membrane.

The newborn patient presented with respiratory distress at 1 h of age and fluctuating tachypnoea during the first 20 h of age. The patient was grunting, hard to comfort and had frequent stools. Nonetheless, the patient had a normal respiratory rate (RR) and oxygen saturation (SaO2), normal blood-sugar levels (lowest value 2.8 mmol/L) and a normal body temperature. Thus, the pediatrician on call assessed that the patient was not tachypneic and clinically stable. No blood test was taken, and no further examinations were made at that point. The patient’s condition was unchanged during the first day. However, at 36 h of age, he was found pale and distressed, whereupon he was admitted to the neonatal unit with a suspected infection.

Upon admittance the patient had a temperature of 36.8°C, Median Arterial Pressure (MAP) 43 mmHg, RR 65/minute, SaO2 100% and capillary refill of 3–5 s (chest-foot). Blood tests showed an elevated C-reactive protein (CRP) 85 mg/L (n.v 0–3), a low white blood count (WBC) of 0.8 × 10^9^/L (n.v 5–25) and normal levels of blood platelets (TPK) 144 × 10^9^ (n.v 85–475). Initial venous blood-gas analysis showed pH 7.25 (7.32–7.43); pCO2 5.8 kPa (5.3–6.6); glucose 2.9 mmol/L (n.v 4.0–6.0); lactate 7.5 mmol/L (n.v 0.5–2.3) and BE −8 mmol/L (n.v −3–3). Benzylpenicillin (100 mg/kg/day) and amikacin (18 mg/kg/day), was given as recommended first-line therapy for early neonatal sepsis. Sepsis work-up was done and the mother’s chart was checked for risk factors for infection. A maternal urinary culture taken, on the day of delivery, showed the growth of *E. coli* with ongoing susceptibility testing. There were no signs of earlier ESBL- *E. coli* colonization or infection in the maternal medical record, since the MDR-marking previously had been removed. Seven hours after admission, the patient developed seizures with electrographic correlates and phenobarbital and later midazolam was given. A lumbar puncture was performed, and cerebrospinal fluid (CSF) analysis showed pleocytosis with WBC of 1639 × 10^6^/L (n.v 0–5), RBC of 125 × 10^6^/L (n.v 0–1), polymorphonuclear leukocytes 781 × 10^6^/L (n.v 0–1), monomorphonuclear leukocytes 858 × 10^6^/L (n.v 0–5), lactate 8.2 mmol/L (n.v 1.1–2.4) and glucose of <0.7 mmol/L. The patient deteriorated into severe metabolic acidosis with pH 7.06 (n.v 7.32–7.43); lactate 15 mmol/L (n.v. 0.5–2.3) and BE −16 mmol/L (n.v −3–3). The patient received mechanical ventilation and antimicrobial therapy was changed to cefotaxime (150 mg/kg/day), ampicillin (300 mg/kg/day) and acyclovir (60 mg/kg/day), according to guidelines with suspicion of meningitis.

A Gram-negative rod grew rapidly in blood (3.7 h) and a few hours later it was found to be an *E. coli*. At that time, cefotaxime was changed to meropenem (40 mg/kg/day). An hour later, it was reported to be an ESBL- *E. coli* strain, susceptible to carbapenems (i.e., imipenem, meropenem and ertapenem, but resistant to cefotaxime, ceftazidime, gentamicin, and ciprofloxacin) according to European Committee on Antimicrobial Susceptibility Testing (EUCAST)’s breakpoints. Thus, the strain was resistant to earlier applied treatment in this case. The next day, the same bacterial strain grew in the child’s cerebrospinal fluid and in the mother’s urine. New blood tests showed an increasing CRP 119 mg/L (n.v 0–3), continuously low WBC 1.6 × 10^9^/L (n.v 5–25) and decreasing TPK 22 × 10^9^ (n.v 85–475). The patient proceeded into to severe respiratory failure despite increased supportive mechanical ventilation. Hypotension was treated with fresh frozen plasma and inotropes, with no effect on urine production. Echocardiography confirmed pulmonary hypertension. Seizures, non-responsive to treatment, increased as the clinical picture of fulminant septic shock developed. Due to poor prognosis, the intensive care was withdrawn from the patient at 3 days of age. This followed an ethical discussion and was in consent with the parents.

## Diagnostic Assessment

Samples of the blood- and cerebrospinal fluid from the child as well as a urine sample from the mother were analyzed. The bacteria grew on blood agar plates at 37°C after 3.7 h and were thereafter typed as *E. coli* with Matrix-Assisted Laser Desorption/Ionization Time of Flight Mass Spectrometry (MALDI-TOF MS). Susceptibility testing of the *E. coli* was performed with disk diffusion, which 2.5 days after the samples had been taken, showed that the strain was ESBL-producing with resistance to cefotaxime, ceftazidime, gentamicin and ciprofloxacin. The bacteria showed susceptibility to carbapenems and amikacin. Epidemiological typing was thereafter performed with Multi-Locus Sequence Typing (MLST), showing that the *E. coli* in all three samples belonged to ST 1193.

## Discussion

An ESBL-*E. coli* that has successfully caused a maternal UTI could be considered a potentially virulent clone and likely to colonize the maternal gut for a considerable amount of time. Maternal intestinal MDR-colonization is not treated with antibiotic prophylaxis. MDR-colonization could be considered a risk factor for early onset MDR-sepsis in and therefore be considered a risk factor for early onset MDR-sepsis in newborn children, particularly when having caused an infection during pregnancy. Awareness of this could lead to a quicker recognition of, and testing for, an infection in these children and increase the chance of correct treatment early on. Early blood-culturing could be considered in these cases. Moreover, it should be highlighted that some ST’s of ESBL- *E. coli* are more virulent than others and therefore at higher risk of causing invasive disease in neonates (3, 4). This case underlines the importance of not falsely removing markings for MDR-colonization from medical records, since it risks causing delay in treatment. In this case, if knowing that the mother previously had been infected with an ESBL-*E. coli*, the antibiotic treatment could have been broadened earlier. However, it might not have changed the fatal outcome for this patient, due to the rapid progress into septic shock.

*E. coli* ST 1193 is a highly virulent strain with increased global spreading, which has until now, been seldom described in neonates. In a recent study of neonatal invasive *E. coli* and their molecular characteristics in China, ST 1193 was the most frequently detected clone and 33% of the *E. coli* ST 1193 was ESBL-producing (14). Further, there are data on a rapid increase in *E. coli* ST 1193 in isolates from young adults in the United States and the first-time prevalence of *E. coli* ST 1193 in neonates in the country was described in 2019 (15). We have, in this case report, described the first known case in Sweden, where a newborn died because of an *E. coli* ST 1193 meningitis. To our knowledge, only one fatal case of neonatal meningitis caused by *E. coli* ST 1193 has globally been described earlier, with a similar fulminant course as in our case (16).

From this case we conclude that studies regarding virulence factors of ESBL-*E. coli* infections in pregnant women and their children are needed to find which newborn babies that could be at risk for severe invasive disease. Such studies might lead to new recommendations in the management of the neonates born to ESBL- *E. coli* colonized mothers, i.e., interventions including intensifying the observations of vital parameters of the newborn in the postnatal ward and a new increased awareness of potential severe invasive neonatal infection. Further, it highlights the risk of delay in diagnosis when a marking for a MDR bacteria is removed from a medical record.

## Data Availability Statement

The original contributions presented in the study are included in the article/supplementary material, further inquiries can be directed to the corresponding author.

## Author Contributions

FO, AL, and VN wrote the first draft of the manuscript. FO and VN coordinated the work around the manuscript. AL and FO extracted the original information from the patients medical records. MF, MK, PE, and CG wrote sections of the manuscript. All authors contributed to manuscript revision, read, and approved the submitted version.

## Conflict of Interest

The authors declare that the research was conducted in the absence of any commercial or financial relationships that could be construed as a potential conflict of interest.

## Publisher’s Note

All claims expressed in this article are solely those of the authors and do not necessarily represent those of their affiliated organizations, or those of the publisher, the editors and the reviewers. Any product that may be evaluated in this article, or claim that may be made by its manufacturer, is not guaranteed or endorsed by the publisher.
